# Long Non-Coding RNAs in Obesity-Induced Cancer

**DOI:** 10.3390/ncrna4030019

**Published:** 2018-08-28

**Authors:** Mabel Yin-Chun Yau, Lu Xu, Chien-Ling Huang, Chi-Ming Wong

**Affiliations:** 1 School of Medical and Health Sciences, Tung Wah College, Hong Kong, China; mabelyau@twc.edu.hk; 2 Department of Medicine, The University of Hong Kong, Hong Kong, China; irisxu@hku.hk; 3 Department of Health Technology and Informatics, The Hong Kong Polytechnic University, Hong Kong, China; cl.huang@polyu.edu.hk

**Keywords:** obesity, cancer, lncRNA, ANRIL, H19, HOTAIR

## Abstract

Many mechanisms of obesity-induced cancers have been proposed. However, it remains unclear whether or not long non-coding RNAs (lncRNAs) play any role in obesity-induced cancers. In this article, we briefly discuss the generally accepted hypotheses explaining the mechanisms of obesity-induced cancers, summarize the latest evidence for the expression of a number of well-known cancer-associated lncRNAs in obese subjects, and propose the potential contribution of lncRNAs to obesity-induced cancers. We hope this review can serve as an inspiration to scientists to further explore the regulatory roles of lncRNAs in the development of obesity-induced cancers. Those findings will be fundamental in the development of effective therapeutics or interventions to combat this life-threatening adverse effect of obesity.

## 1. Introduction

Body weight increases when long-term energy intake exceeds energy expenditure, which triggers the increase of energy storage as fat in our body. Increase in body weight does not only enhance the risk of diabetes and cardiovascular diseases, but also contributes to several types of cancers [1,2] and diminishes survival of cancer patients [3]. It was estimated that about 20% of cancer cases are caused by obesity [4]. Our understanding of the interrelationships between obesity and cancer risk have significantly improved in the last century [5]. Several hypotheses were proposed to explain why obesity may foster or promote cancers [6,7,8]. The generally accepted hypotheses are included below.

### 1.1. Hyperinsulinemia

Insulin resistance is a common characteristic of obese people [9]. Insulin resistance stimulates the pancreas to produce more insulin leading to hyperinsulinemia. A chronic high level of insulin can be harmful to our body because of its mitogenic and anti-apoptotic effects [10]. Elevated circulating insulin level potentially favors the growth and aggressiveness of cancers. In addition, hyperinsulinemia upregulates the expression and activity of insulin-like growth factor-1 (IGF-1) [11]. Insulin shares significantly high homology with IGF-1 and can interact with IGF-1 receptor (IGF-1R) [4]. As a high level of IGF-1 and dysregulation of IGF-1R signaling are also associated with tumor development [12], it is necessary to evaluate the administration level of native insulin or insulin analogue in terms of reducing cancer risk [7,13].

### 1.2. Dysregulation of the Adipokine Expression

The main role of adipose tissue is not only energy storage [14]. Adipose tissue is also an important endocrine organ [15] producing hundreds of cytokines, the adipokines. Secretion profiles of adipokines are affected by the size, metabolic compositions of adipocytes [16], and their cellular populations [17]. For example, leptin is an adipokine predominantly produced by adipocytes that inhibits appetite [18]. Similar to the situation of insulin resistance, leptin resistance is also commonly found among overweight people. Moreover, leptin resistance is believed to be one of the leading drivers of weight gain [18]. Anti-apoptotic and mitogenic effects of leptin have been demonstrated on different cancer cell lines [19]. From reports, the significantly high circulating level of leptin is associated with enhanced cell proliferation and increased risks of cancers in the leptin resistance in obese subjects [20,21,22].

### 1.3. Hypoxia

Cells adjacent to the blood vessels are exposed to relatively higher O_2_ level than the cells away from the blood vessels. As fat accumulates in the adipose tissues during the development of obesity, the “well” vascularized adipocytes may become “poorly” vascularized [23]. Low oxygen tension (hypoxia) in adipose tissues triggers necrotic cell death and inflammation [24]. Hypoxia stimulates a complex cell signaling network such as hypoxia-inducible factor 1 (HIF-1) [25]. HIF-1 is a heterodimeric transcription factor, composed of HIF-1α (or its analogs HIF-2α and HIF-3α) and HIF-1β [26]. HIF-1 is the key of oxygen sensing mechanism in mammalian cells and plays a crucial role in the adaptation to hypoxic stress of the cancer cells [27]. HIF-1α is constitutively transcribed and synthesized independently from O_2_ concentration, but HIF-1α will be degraded quickly under normoxic conditions [26]. In other words, the stability of HIF-1α increases under hypoxic conditions. Recent studies demonstrated the correlation of high-level HIF-1 associated with tumor metastasis and poor prognosis in patients [28]. Increased adipose HIF-1α protein was detected with obesity-associated factors [29], which enhanced cancer progression [30].

### 1.4. Chronic Inflammation

Inflammatory pathways is an approach of host defense, but chronic low-level inflammation is suggested to cause cancers [31]. It is estimated that about 20% of cancers in humans are contributed by chronic inflammation [32]. Under inflammatory conditions, free radicals are produced from inflammatory cells and cause DNA damage [33]. A number of mechanisms of obese-related chronic inflammation and cancer were proposed. For instance, various adipokines are proven to be involved in the inflammatory processes [34]. However, the upregulation of adipokines is not totally associated with the size and number of adipocytes. In contrast, the expression level of anti-inflammatory adipokine, adiponectin, is decreased in obese subjects [35]. This net change of expression between proinflammatory and anti-inflammatory adipokine contributes to local and systemic inflammation in those who are obese [36].

In addition to the contribution of adipokines in inflammation, emerging evidence demonstrated that changes in gut microbiota composition and increased intestinal permeability of obese subjects promote the uptake of endotoxin (lipopolysaccharides, LPS) produced from intestinal microorganisms [37]. The systemic elevations of gut-derived LPS activate the pattern recognition receptors and initiate inflammatory cascades [38]. This model is supported by the increase in circulating endotoxin among obese individuals, and the decrease of LPS in individuals following weight loss and/or gut modulation therapy [37]. Dysbiosis or imbalance in gut microbiota has been associated with the pathogenesis of obesity [39]. The precise mechanism of obesity-associated inflammation is still being explored [37].

Other than the hypotheses mentioned above, genetic and epigenetic factors also play an important role in obesity-related cancers [40,41]. Diets and environmental factors can cause significant epigenetic changes [42] and affect the expression of genes, including genes that contain the sequences of long non-coding RNAs (lncRNAs). Recent studies demonstrated that the expression profiles of lncRNAs were found to be significantly different in obese and non-obese human subjects [43,44]. Moreover, numerous functional lncRNAs are involved in lipid metabolism and adipogenesis [45,46]. In this mini review, we summarize the emerging evidence of a link between lncRNAs, obesity, and cancers. The emerging findings for three well-known oncogenic lncRNAs (namely, antisense non-coding RNA in the INK4 locus (*ANRIL*), *H19*, and *HOX* transcript antisense RNA (HOTAIR)) and their potential roles in obesity-induced cancers are discussed. Understanding the potential roles of lncRNAs will provide insights to further develop prevention and treatment strategies for obese-related cancers.

## 2. lncRNAs in Cancer and Energy Metabolism

Most long lncRNAs synthesized by RNA polymerase II (RNA Pol II) are 5′ capped, spliced, and polyadenylated by a similar transcriptional machinery to messenger RNA (mRNA) [47]. By definition, lncRNAs are transcripts longer than 200 nucleotides (nt), most of which are not translated into protein [48]. Interestingly, it was recently reported that protein translation indeed exists in about 40% of lncRNAs [49]. With the advancement of sequencing technologies and computation methods for transcriptome assembly, a large number of lncRNAs have been identified. According to the Encyclopedia of DNA Elements (ENCODE) project, more than 28,000 lncRNAs are encoded from 16,000 genes in human [50]. lncRNAs are implicated in a variety of biological processes and diseases, most notably in cancers [47,50,51]. Early studies focus on the regulatory role of lncRNAs on gene expression at transcriptional or post-transcriptional levels under pathophysiological conditions [47,52,53]. As several lncRNAs have been found in exosomes and are thus protected from endogenous RNases, they can be detected in body fluids such as blood and urine. In this regard, secreted lncRNAs may become valuable biomarkers for many diseases including cancers [54].

Recent studies demonstrated that many lncRNAs regulate adipogenesis [55] and lipid homeostasis [56]. Systematic transcriptome analysis was performed to evaluate the significance of lncRNAs in metabolic homeostasis by comprehensively profiling lncRNAs in key metabolic organs under different metabolic conditions. The findings demonstrated that many lncRNAs are regulated by nutrient factors and metabolic hormones [57]. Differentially expressed circulating lncRNAs were also reported in obese patients using microarray analysis [43]. Given the role of lncRNAs in metabolic homeostasis, the design of lncRNA-based therapies could be considered. Screening strategy using pharmacological compounds for the treatment of obese-related diseases can be further utilized [44]. Interestingly, a number of cancer related lncRNAs are dysregulated/co-expressed in obesity, suggesting that obesity-associated lncRNAs may promote cancers. Here, we summarize the emerging findings for three well-known oncogenic lncRNAs (namely, ANRIL, H19, and HOTAIR) and discuss their potential roles in obesity-induced cancers (Table 1).

### 2.1. Antisense Non-Coding RNA in the INK4 Locus

ANRIL (antisense non-coding RNA in the INK4 locus; also known as CDKN2B-AS1) is transcribed as a ~3.8 kb nucleotide-long lncRNA from the short arm of human chromosome 9 near INK4/ARF (INK4B–ARF–INK4A) locus [97]. INK4/ARF locus encompasses three important tumor suppressors, p14(ARF), p15(INK4b), and p16(INK4a) [98]. p15(INK4b) and p16(INK4a) are CD1/6 inhibitors that activate pRb, whereas p14(ARF) is an Mdm2 inhibitor that activates p53 [98]. The genes at this locus cause cell cycle arrest. Deletion, mutation, or transcriptional silence of the genes at this locus lead to 30–40% of human tumors [99].

The expression of ANRIL and the tumor suppressor genes at INK4/ARF locus is highly coordinated through a shared bidirectional promoter [100]. ANRIL is also one of the most up-regulated lncRNAs in cancers [101]. A recent study demonstrated that the expression of ANRIL is transcriptionally induced by DNA damage, especially at the late stage of the DNA damage response (DDR) [102]. DNA damage induces the expression of the genes (*p14*, *p15*, and *p16*) at the INK4/ARF locus. ANRIL acts as homeostatic regulator to escape from the DDR activity by downregulating the expression of *p14*, *p15*, and *p16* in the INK4/ARF locus [102]. In precancerous lesions, the aberrant expression of ANRIL leads to genomic instability by blocking the control of the DDR mechanism. The mechanism is further supported by a recent study, demonstrating that the ATM-E2F1 signaling pathway induces ANRIL overexpression [72].

In addition, ANRIL can directly interact with and recruit polycomb repressive complex-2 (PRC2) complex to repress the expression of *p15(INK4b)* [99] and genes at the CDKN2A/B locus [97]. It is linked to poor prognosis of cancers by silencing this tumor suppressor locus. Insulator binding protein CTCF (also known as 11-zinc finger protein or CCCTC-binding factor) is one of the key transcription factors that regulates the expression of ANRIL and these three tumor suppressor genes at the INK4/ARF locus by modulating the chromatin architecture [103]. The recruitment of CTCF is dependent on the differential DNA methylation [104]. The CpG methylation of DNA disrupts the binding of CTCF to DNA [105], which contributes to gene silencing at the locus permanently [103].

Numerous polymorphisms located at the ANRIL locus have been highly associated with increased risk of diabetes and cardiovascular diseases (Table 2) [97,106,107]. Interestingly, the lower level of CpG methylation within the promoter of ANRIL at birth is associated with increased cardiovascular risk [108] and adiposity [109] at later childhood. This association derived from promoter methylation on ANRIL and adiposity was also determined in human tissues at various developmental stages [109]. As mentioned above, CpG methylation at the promoter of ANRIL also affects the expression of suppressor genes at the INK4/ARF locus. Further studies are required to reveal the important functions of ANRIL, in particular the incidence of cancers in obese children.

Furthermore, a recent study on genome-wide expression profiling demonstrated that downregulation of ANRIL transcripts containing exon 13 is correlated with the decrease in the expression of ADIPOR1, VAMP3, and C11ORF10 [117]. These observations are associated with various metabolic traits via glucose and fatty acid metabolism [117]. This indicated that ANRIL might have a potential function in regulating energy metabolism. In addition, further determining the differentially expressed combination of exons in different conditions is required for multi-exonic lncRNAs [118,119].

### 2.2. H19

**Figure 1:**
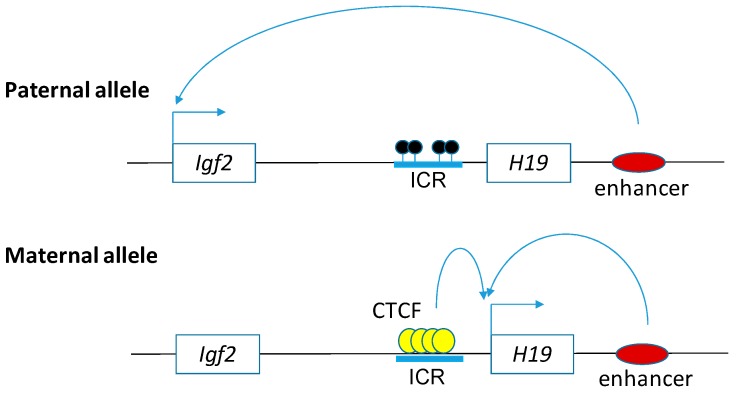
The epigenetic state of imprinting control region on the *Igf2*-*H19* locus determines the expression pattern. Regulation of maternal and paternal expression in the *Igf2*-*H19* imprinted domain is controlled by genomic DNA methylation. The open boxes represent the genes *Igf2* and *H19*, and the blue boxes represent the imprinting control region (ICR). The close lollipops represent methylated CpG islands. The yellow and red circles represent the CCCTC binding factor (CTCF) insulator protein and enhancer, respectively. The arrows from the boxes indicate expression of the genes. *Igf2* and *H19* genes are activated by the shared downstream enhancer, and their activations are dependent on the DNA methylation of the ICR. CCCTC binding factor (CTCF) is recruited to unmethylated ICR on the maternal allele that promotes the enhancer to activate the expression of *H19* gene, but not of *Igf2* gene. In contract, on paternal allele, ICR is hypermethylated that prevents the binding of CTCF to ICR. The overall outcomes are that the expression of *H19* is repressed, but the expression of *Igf2* is induced, from the paternal allele.

H19 is encoded from a 2.7 kilobases gene, which is maternally expressed and paternally imprinted, and is located closely to the telomeric region of chromosome 11 (Figure 1) [120]. H19 was first identified as one of the most abundant RNAs in the developing mouse embryo, and its expression is repressed in all murine tissues except skeletal muscle [121]. That is why *H19* was first named ASM (adult skeletal muscle) [122]. The nucleotide sequence of rodent and human *H19* is evolutionarily conserved [121]. During mammalian development, *H19* expression is predominantly regulated by DNA methylation at imprinting control regions on its promoter [123]. Aberrant relaxation of imprinted *H19* has been detected in a wide variety of cancers [124]. As *H19* play a crucial role in embryogenesis and controls the expression of two major pluripotency factors—Oct4 and Sox2 [125], it promotes cancer stemness [126], which is associated with poor prognosis in cancer patients [127].

Most studies indicated that overexpression of *H19* is associated with tumorigenesis (Table 1). Recent meta-analyses showed that the genetic variants of *H19* (e.g., allele rs2839698) exhibited a significantly higher risk of developing cancer [128]. Poor overall survival could be predicted by high levels of H19 expression [129,130]. Therefore, *H19* was proposed to serve as a biomarker for poor prognosis in various cancers with different types of predictive factors and clinicopathological features [130]. Moreover, the inhibition of H19 represents a potential candidate for cancer therapies [131].

Two major mechanisms of *H19* in cancers were proposed. Firstly, *H19* is a developmental reservoir of miR-675 that suppresses the expression of many tumor suppressors [125,132] (Table 3). Secondly, *H19* functions as a modulator by binding directly to microRNAs (miRNAs) or proteins [125]. *H19* acts as a molecular sponge to modulate the availability of miRNAs such as let-7 [133,134]. Let-7 was first identified as a key developmental regulator in nematode. The sequences of let-7 are highly conserved across species. Based on their expressions during developmental processes in vertebrates, let-7 is one of the most abundant miRNAs in adult mammalian tissues and acts as a tumor suppressor by promoting termination of differentiation [135]. There is growing evidence that many human cancers have deregulated let-7 expression, and restoring the let-7 expression may be a useful therapeutic approach in cancers [136]. Interestingly, a recent study demonstrated that let-7 also regulates H19 expression via the RNA-binding pluripotent stem cell factor LIN28 [137]. LIN28 is also a let-7 target and can drive tumor initiation and progression [138]. Intriguingly, LIN28 also blocks mature let-7 production [137]. By this negative feedback loop, breast cancer stem cell maintenance is promoted by H19/let-7/LIN28 axis [137].

Previous studies have demonstrated that the LIN28/let-7 axis regulates glucose metabolism [150,151,152]. In brief, to upregulate the bioenergetic state of cells, glucose uptake and increasing the activities of both glycolysis and mitochondrial oxidative phosphorylation can be enhanced by reactivation of LIN28 and suppression of let-7 [138]. In addition, a recent study demonstrated that the expression of H19 is regulated by let-7, which is important for the muscle glucose metabolism [153]. As mentioned above, H19 is uniquely and highly expressed in muscle at all the studied ages. Therefore, exploring the physiological function of H19 in muscle will be of fundamental importance to various human diseases. Depletion of H19 impaired insulin sensitivity of muscle cells, which correlates with impaired glucose homeostasis in human and mouse [153].

Besides binding to miRNAs, H19 RNA also interacts with proteins. For example, H19 binds to hnRNP U, disrupts the hnRNP U-actin complex, and hence inhibits RNA Pol II-mediated transcription [154,155]. Blocking the interaction of hnRNP U and actin was proposed to have a crucial effect on the fetal liver development [84]. In addition, H19 can alter the tumor suppressor miR-200 by increasing histone acetylation via the association with the protein complex hnRNP U/PCAF/RNAPol II [156].

Differentially methylated and imprinted control regions were found in the *H19* promotor [157]. Methylation of the *H19* promoter is negatively correlated with H19 expression. Under normal circumstances, in the offspring cell, the paternal copy of *H19* is methylated and silent, but the maternal copy is hypomethylated or unmethylated and expressed [158]. In addition, like many other imprinted control genes, epidemiologic studies have demonstrated associations between environmental exposures and the expression of H19 [159]. To show the potential effects of maternal and paternal pre-conceptional over-nutrition, newborns of obese parents who have altered DNA methylation patterns at imprinted genes were reported recently [160]. The methylation percentages in a differentially methylated region (DMR) of *H19* may be associated with childhood obesity in children [161,162]. Increased expression of H19 and miR-675, as well as altered methylation of the *H19* imprinting control region, are associated with a low fat-free mass index in patients with chronic obstructive pulmonary disease [163]. The association among the altered methylation of the *H19* imprinting control region and H19/miR-675 expression in obesity-induced cancers remains to be explored.

### 2.3. HOTAIR

HOTAIR (*HOX* transcript antisense RNA) is a ~2.2 kb nucleotide-long lncRNA, which transcribed in an antisense manner with respect to the *HOXC* genes, located on chromosome 12 [164]. *HOX* genes encode for regulatory transcription factors during embryogenesis [165]. Previous studies have demonstrated that *HOX* gene products also play significant roles in the development of cancers [166]. Numerous antisense lncRNAs regulate the associated protein coding genes in cis manner. However, although *HOTAIR* gene is encoded in the antisense-strand of *HOXC* genes, there is evidence that supports the fact that HOTAIR does not affect the expression of genes at the *HOXC* locus [167]. Similar to ANRIL, HOTAIR can affect chromatin state by interacting with PRC2 [168]. Alternatively, HOTAIR can repress the expression of genes at HOXD cluster via PRC2 complex [167,169]. HOTAIR is suggested to be a potential oncogene and is expressed in cancerous tissues higher than non-cancerous tissues (Table 1). It has been shown to have a significant impact on the tumor cell viability, proliferation, and invasion [168]. As its expression can be used to predict the metastatic progression and overall survival, HOTAIR was proposed as a prognostic biomarker in different types of cancers [170,171].

It is generally believed that the global obesity pandemic is mainly a result of the high-caloric food and sedentary lifestyle [172]. A recent study demonstrated that a sedentary lifestyle further increases circulating exosomal HOTAIR in obese subjects, but not in lean subjects [173]. HOTAIR was expressed in gluteal adipose and a large increase in HOTAIR expression could induce differentiation in abdominal preadipocytes [174]. Scanning electron microscope (SEM) analysis further demonstrated that the gluteal adipose tissue was active in exosome biogenesis and secretion [173]. The induction of HOTAIR expression in gluteal fat upon squeeze is transcriptionally regulated via NFκB [173]. The same research team also found that sedentary lifestyle promoted gluteal-femoral fat to secrete exosomal HOTAIR that promotes intestinal cell proliferation [173]. Sedentary behavior increases the risk of certain cancers [175,176]. This study proposed a possible explanation for the linkage between obesity, sedentary lifestyle, and colorectal cancers via lncRNA HOTAIR. It is worthy to further explore whether sedentary lifestyle-induced exosomal HOTAIR from adipose tissues also promotes other obesity-related cancers.

## 3. Conclusions

**Figure 2:**
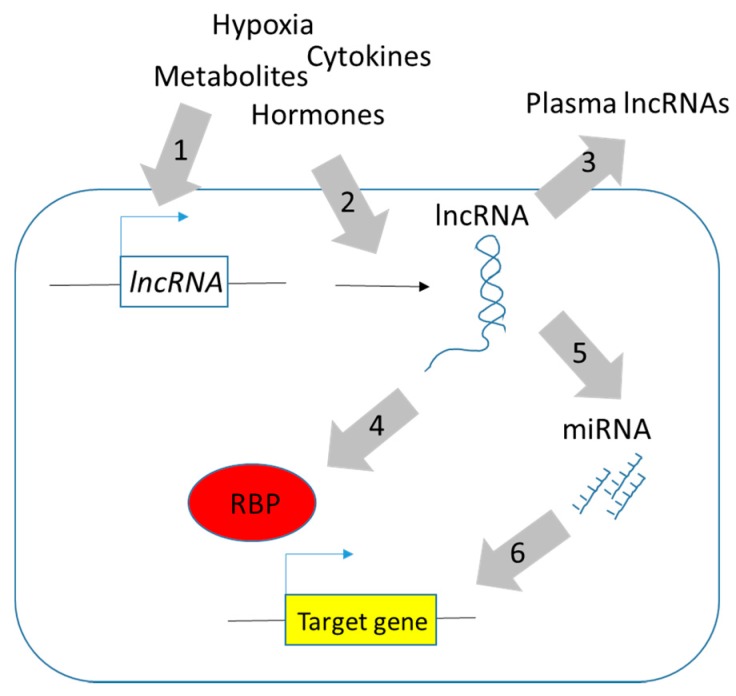
Proposed roles of long non-coding RNAs (lncRNAs) in obese-induced cancers. Many obese-related physiological changes such as nutrient availability, oxygen level, inflammatory cytokines, and metabolic hormones may affect the expression level (1) and post-transcriptional processing (2) of endogenous lncRNAs. Many lncRNAs can be transported to circulation and may serve as biomarkers or molecular diagnostic applications (3). Whether or not the exogenous lncRNAs contribute to the cancer progression is just emerging. In contrast, there is plentiful evidence showing that endogenous lncRNAs can regulate gene expression by diverse mechanisms. lncRNAs may act as scaffolds or molecular decoys, which directly interact with RNA binding proteins (RBPs; red circle) such as transcription factors and chromatin-modifying complexes to regulate the expression of proto-oncogenes and/or tumor suppressor genes (4). lncRNAs may act as endogenous sponges regulating gene expression via modulating microRNAs (miRNAs) availability (5 and 6).

A large number of epidemiological and mechanistic studies link obesity to the increased risk of and acceleration of the progression of several types of cancer. New hypotheses, such as kynurenine pathway implicated in both obesity and cancer, were proposed. In this review, we have briefly summarized the recent evidence to explore the potential contribution of three well-characterized oncogenic lncRNAs to obesity-induced cancers. The evidence shown here is mainly based on correlation and additional in vivo studies. Many oncogenic lncRNAs have been identified in recent years. Whether their expression level can be also regulated by nutrient availability and obese-related physiological changes remains to be investigated. In addition to the association studies, further genetic manipulation and in vivo evaluation are required to verify the actual functions and molecular mechanisms of those lncRNAs (Figure 2). As the expression of lncRNAs affected by epigenetic alterations are potentially reversible changes, these findings will offer attractive and promising strategies for therapeutic intervention.

## Figures and Tables

**Table 1:** Summary of the recent findings on antisense non-coding RNA in the INK4 locus (*ANRIL*), *H19*, and *HOX* transcript antisense RNA (HOTAIR) in various human obesity-induced cancers.

Type of Cancer	*ANRIL*	*H19*	*HOTAIR*
Endometrial cancer	Upregulated [58]	Upregulated [59]	Upregulated [60,61]
Esophageal adenocarcinoma	Upregulated [62]	Upregulated [63,64]	Upregulated [65,66]
Liver cancer	Upregulated [67,68]	Upregulated [69], Downregulated [70]	Upregulated [71]
Pancreatic cancer	Upregulated [72,73]	Upregulated [74]	Upregulated [75,76]
Colorectal cancer	Upregulated [77,78]	Upregulated [64,79], Downregulated [80]	Upregulated [81,82]
Gallbladder cancer	Upregulated [83]	Upregulated [84,85]	Upregulated [86]
Breast cancer	Upregulated [87]	Upregulated [88]	Upregulated [89]
Ovarian cancer	Upregulated [90]	Upregulated [91]	Upregulated [92]
Thyroid cancer	Upregulated [93]	Downregulated [94]	Upregulated [95,96]

**Table 2:** Single-nucleotide polymorphisms (SNPs) in ANRIL locus associated with diabetes and cardiovascular diseases.

SNP-ID	Related Diseases	Remarks	References
rs10757278	Myocardial infarction		[110]
rs2891168	Coronary artery disease	G-allele was associated with lower triglyceride level	[111]
rs10811661	Type 2 diabetes		[111,112]
rs10965215 and rs10738605	Myocardial infarction		[113]
rs10757274 and rs1333042	Coronary artery disease		[114]
rs10757278	Major adverse cardio-vascular event (MACE) in patients starting on hemodialysis		[115]
rs564398	Type 2 diabetes	Reduced β-cell proliferation	[116]

**Table 3:** Targets of miR-675.

Targeted mRNA	Targeted Region	Related Cancer or Diseases	References
FADD	3′-UTR	Gastric cancer	[139]
PTEN	3′-UTR	Restenosis	[140]
Vitamin D receptor	3′-UTR	Colon cancer	[141]
REPS2	3′-UTR	Esophageal squamous cell carcinoma	[142]
RUNX1	3′-UTR	Gastric cancer	[143]
TWIST1	3′-UTR	AFP-secreting hepatocellular carcinoma	[144]
Retinoblastoma	3′-UTR	AFP-secreting hepatocellular carcinoma, Colorectal cancer, glioma	[144,145]
CALN1	3′-UTR	Gastric cancer	[146]
c-Cbl	coding sequence	Breast cancer	[147]
Cbl-b	coding sequence	Breast cancer	[147]
TGFBI	3′-UTR	Prostate cancer	[148]
Cadherin 13	3′-UTR	Glioma development	[149]

Abbreviations: 3’UTR: 3’ untranslated region; AFP: α-fetoprotein.

## Abbreviations

3’UTR	3’ untranslated region
AFP	α-fetoprotein
ANRIL	antisense non-coding RNA in the INK4 Locus
DMR	differentially methylated region
HOTAIR	HOX transcript antisense RNA
HOX	Homebox
IGF-1	insulin-like growth factor-1
lncRNAs	long noncoding RNAs
LPS	lipopolysaccharides
PRC2	polycomb repressive complex-2
RNA Pol II	RNA polymerase II
